# An educated guess — Distance estimation by surgeons

**DOI:** 10.1016/j.sopen.2020.04.001

**Published:** 2020-04-30

**Authors:** R. Gregory Conway, Natalie O'Neill, Jessica Brown, Stephen Kavic

**Affiliations:** a University of Maryland, Department of Surgery, Baltimore, MD, USA; b University of Maryland, Department of Epidemiology and Public Health, Baltimore, MD, USA

## Abstract

**Introduction:**

Estimating distance is a common task in surgery, yet development of distance estimation ability receives little attention in surgical training. Although the Small bites versus large bites for closure of abdominal midline incisions (STITCH) trial reinforced the importance of suture spacing by demonstrating reduced incisional hernia incidence in placement of 5-mm fascial sutures over 1 cm, we hypothesize that neither trainee nor attending surgeons possess the ability to estimate these distances with accuracy.

**Methods:**

We distributed a 4-question distance estimation exercise and a 6-question survey to resident and attending surgeons at a single academic medical center. The mean and the absolute error were compared using a *t* test.

**Results:**

Most participants were trainees (44 vs 16 attendings, N = 60), and 27% used the metric system prior to undergraduate studies. The mean absolute errors for 5-mm and 1-cm mark placement were 1.40 and 2.07 mm, respectively. The 5-mm mark placement estimates ranged from 2.01 to 11.69 mm, and the 1-cm estimates ranged from 4.82 to 19.19 mm. There was no statistically significant difference in the estimates or absolute errors between trainees and attendings (5 mm P = .202; 1 cm P = .302).

**Conclusion:**

These findings suggest that estimation of distance is a challenge, and development of this fundamental skill during surgical training may have important clinical consequences.

## INTRODUCTION

The development of surgical skills during residency training follows a stepwise progression based on the resident's level of experience, demonstrated competence, and case complexity. Despite the presence of this formal training paradigm, skill development often relies on fundamental abilities that are learned early in life. One such fundamental skill that trainees are expected to possess is the ability to estimate short distances, and residents are often requested to perform this task intraoperatively. Generally, length estimation is used when cutting a suture with an appropriate length tail above the knot; determining the spacing of sutures; or choosing an appropriate-sized mesh, conduit, or cannula. Although the attending surgeon is the arbiter for deciding the “correctness” of the trainee's estimate, incongruence between attending and trainee estimates can lead to frustration for the trainee and erosion of the attending's confidence in the trainee's abilities.

Techniques for accurate length estimation have existed for millennia, with the ancient Egyptians developing a measurement system based on anthropomorphic references [1]. Although imprecise, human-body references have the advantage of being nearly ubiquitous and are applicable in many scenarios. In modern times, length estimation is taught to Boy Scouts for the purpose of wilderness exploration and survival skills [2], as well as in military education. These systems rely on comparison to a known reference as a means to increase accuracy.

Despite these existing techniques of measurement estimation, translation of similar techniques to the operating room is not commonplace. Instead, many surgeons rely on their own perception of the distances to be estimated without performing any training to assist in self-calibration. To evaluate the ability of surgeons to estimate short distances, we developed and distributed a survey with several length estimation tasks to attending surgeons and surgical trainees at a single institution. We hypothesize that neither attendings nor trainees possess the ability to estimate short distances with accuracy.

## METHODS

**Fig 1:**
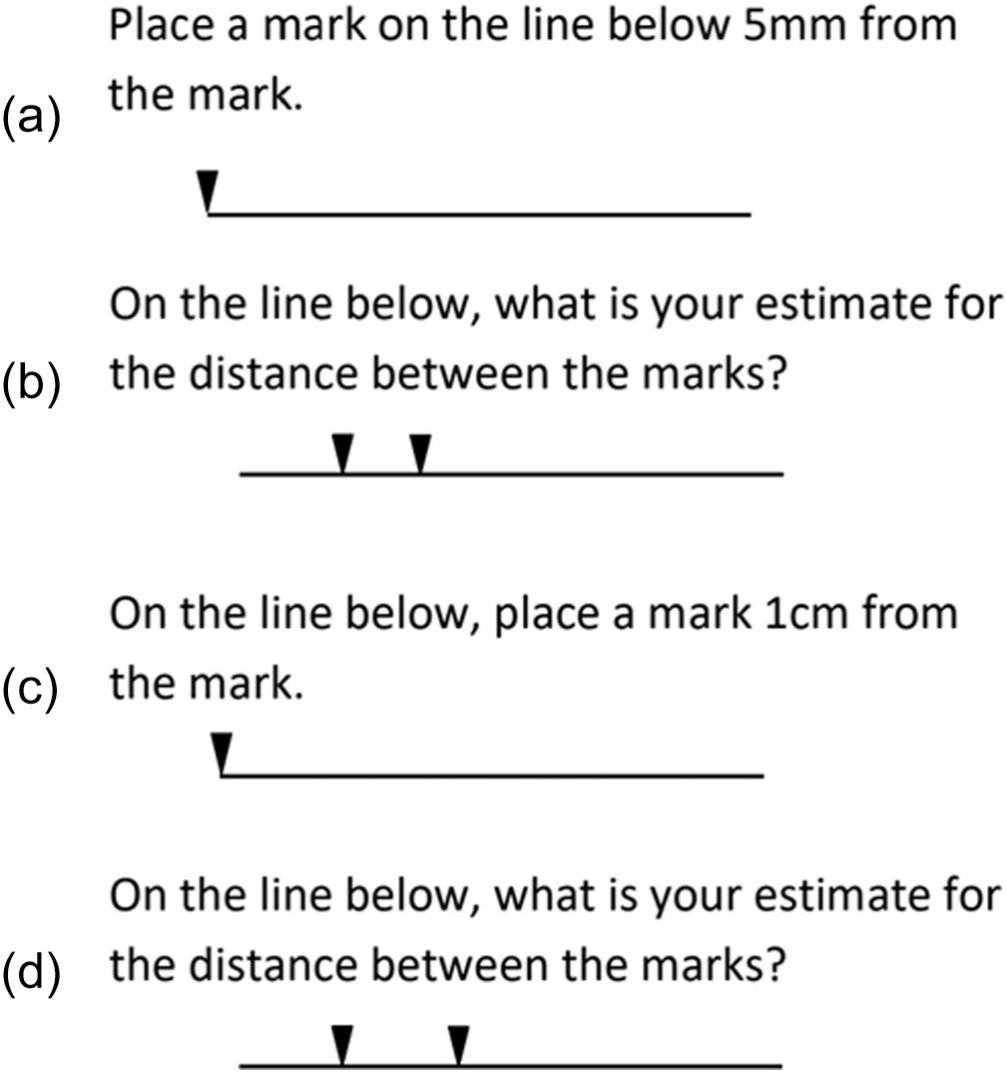
Measurement tasks. Panels A and C require placement of a line at the specified distance. B, The 6-mm distance between marks. D, The 9-mm distance between marks.

After receiving an exemption from review from the University of Maryland Institutional Review Board, a survey was distributed to resident and attending surgeons at our medical center. A novel survey was developed at our institution and consisted of 10 questions in total, with 4 of the questions pertaining to measurement tasks. Participants were given a single attempt to complete the survey. Specifically, participants were asked to place a mark 5 mm from an indicator on a line, to place a mark at 1 cm from an indicator on a line, and to select the distance between 2 marks (6- and 9-mm spacing) from multiple choice options (Fig 1). The multiple choice options for the 6- and 9-mm spacing tasks ranged from 4 to 12 mm by 1-mm increments. The remaining questions related to training level, years of operative experience, number of laparotomy closures per month, and preferred suture spacing for laparotomy closure. To assess the participant's familiarity with the metric system and US customary system of units, the preferred units of measure used prior to undergraduate studies and the preferred units of measure used currently were asked. Specific survey questions are provided in Appendix A. The survey questions underwent expert review and were subsequently pretested by 4 nonsurgical medical professionals to ensure survey clarity. The surveys were printed on standard 8.5 × 11” paper, and the length estimation questions were measured with a digital Vernier caliper (Mitutoyo 500-196-30, 0.01-mm resolution) to ensure that page scaling was accurate. Surveys were distributed to surgery residents at the biannual Residency Advance conference and to attending surgeons at the weekly morbidity and mortality conference. The survey was distributed once at each of these conferences. Return of the survey to the study coordinators was considered as consent to include the individual's responses in the study.

Responses on the returned surveys were recorded into a spreadsheet (Excel 2010; Microsoft, Redmond, WA). The length estimates for the 5-mm and 1-cm marks were measured with a digital Vernier caliper to the nearest 0.01 mm. The absolute error from the correct measurement was calculated for each of the 4 measurement tasks and was included as a separate variable in the data set. Univariate statistics were computed for each study variable and for the absolute error variables. Bivariate statistics were calculated based on training level, years of operative experience, laparotomy closure experience, and measurement system preference, with *P* values calculated using *t* tests. The equivalence of variance between groups was computed using Levene tests. All statistical calculations were performed using SAS software (version 9.4; SAS, Cary, NC).

## RESULTS

Forty-five surveys were distributed at the Residency Advance conference, with 42 completed surveys returned. At the morbidity and mortality conference, 28 surveys were distributed, with 18 completed surveys returned. From the 60 completed survey responses received, 44 were residents and 16 responses were from attending surgeons. Twenty-five respondents were junior residents (PGY 1, 2, or research), whereas 19 were in their senior years (PGY 3–5). Among attending surgeons, 9 performed more than 5 laparotomy closures per month (56.25%), and 75% had more than 10 years of operative experience (*n* = 12). Nine respondents preferred a 5-mm by 5-mm suture spacing for laparotomy closure (15%), 21 preferred a 1-cm by 1-cm suture spacing (35%), and 26 reported a suture spacing between 5 mm by 5 mm and 1 cm by 1 cm (43.3%). Fifty-four respondents report a preference for the metric system of length measurement currently (90%), with 16 reporting using the metric system prior to undergraduate studies (26.7%). Table 1 summarizes these results.

**Table 1:** Participant characteristics

		*Overall* *[*N *= 60]*	*Resident* *[*n *=* *44]* n *(%)*	*Attending* *[*n *=* *16]* n *(%)*	P *value*
Operative Experience	1–5 y	40	13 (88.6)	1 (6.2)	< .001
	> 5 y	20	5 (11.4)	15 (93.8)	
Laparotomy experience	1–5 per month	35	28 (63.6)	7 (43.8)	.092
	> 5 per month	25	16 (36.4)	9 (56.2)	
SI units prior to Undergraduate		16	11 (25.0)	5 (31.3)	.224
Current SI unit preference		54	40 (90.9)	14 (87.5)	.325

*P* values computed from *χ*^2^ or Fisher exact test, as appropriate.

**Fig 2:**
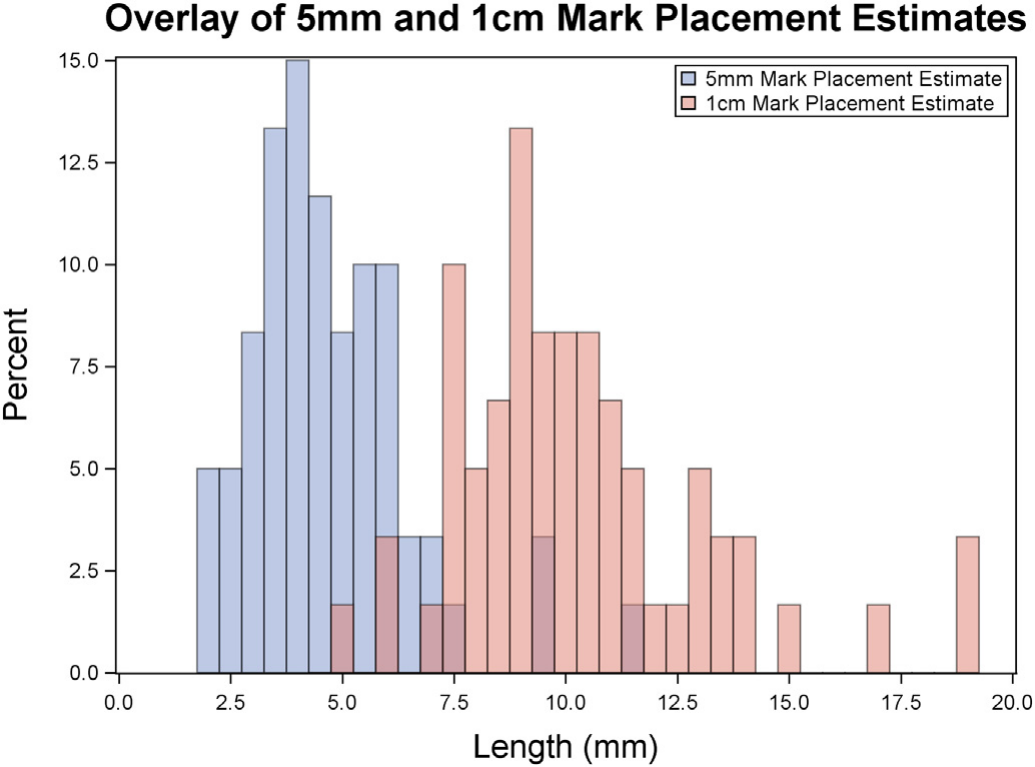
Histogram of 5-mm and 1-cm mark placement estimates.

A histogram of distance estimation responses is shown in Fig 2, and a summary of these responses is shown in Table 2. For the 5-mm mark placement task, the mean distance was 4.72 mm, ranging from a minimum of 2.01 mm to a maximum estimate of 11.69 mm. Responses for the 1-cm mark placement task ranged from 4.82 to 19.19 mm, with a mean response of 10.23 mm. The mean absolute error for the 1-cm mark placement task was 2.07 mm. The 6- and 9-mm estimation tasks yielded means slightly above the correct value, at 6.32 and 9.43 mm, respectively. The mark placement and distance estimation tasks, as well as the absolute errors of these tasks, were compared between attending and resident participants, and no statistically significant differences were detected (all *P* ≥ .149; Table 2).

**Table 2:** Measurement estimates overall and by resident or attending status

	*Overall* *Mean (SD, range)*	*Residents* *Mean (SD, range)*	*Attendings* *Mean (SD, range)*	t *test* P *value*	*Levene test* P *value*
5-mm mark placement	4.72 (1.83, 2.01–11.69)	4.90 (1.80, 2.01–11.69)	4.22 (1.86, 2.03–9.69)	.202	.969
Absolute error	1.40 (1.19, 0.01–6.69)	1.32 (1.22, 0.01–6.69)	1.63 (1.13, 0.35–4.69)	.369	
1-cm mark placement	10.23 (2.83, 4.82–19.19)	10.46 (2.56, 7.19–19.19)	9.60 (3.47, 4.82–18.87)	.302	.283
Absolute error	2.07 (1.92, 0.09–9.19)	1.85 (1.81, 0.09–9.19)	2.66 (2.16, 0.24–8.87)	.150	
6-mm distance estimation	6.32 (1.60, 4.0–10.0); median 6.0, mode 6.0	6.14 (1.52, 4.0–10.0); median 6.0, mode 5.0	6.81 (1.76, 4.0–10.0); median 7.0, mode 6.0	.149	.467
Absolute error	1.28 (0.99, 0.0–4.0)	1.23 (0.89, 0.0–4.0)	1.44 (1.26, 0.0–4.0)	.473	
9-mm distance estimation	9.43 (1.84, 4.0–12.0); median 10.0, mode 10.0	9.43 (1.73, 4.0–12.0); median 10.0, mode 10.0	9.44 (2.16, 5.0–12.0); median 9.5, mode 9.0	.992	.373
Absolute error	1.47 (1.17, 0.0–5.0)	1.39 (1.10, 0.0–5.0)	1.69 (1.35, 0.0–4.0)	.383	

Responses were classified based on the measurement system used prior to undergraduate studies. Those respondents who used the metric system of units during these years of early education were found to have greater errors in the 5-mm mark placement, 1-cm mark placement, and 6-mm distance estimation tasks when compared to respondents who did not use the metric units prior to undergraduate studies (*P* = .011, .042, and .028, respectively; Table 3). Those respondents who report using the metric system currently had a larger absolute error for the 5-mm mark placement task (1.46 vs 0.90 mm, *P* = .022) and the 1-cm mark placement task (2.19 vs 0.97 mm, *P* < .001). No other estimation tasks for the current measurement system showed a statistically significant difference. There were no statistically significant differences in any estimation task for frequency of laparotomy closure or years of experience.

**Table 3:** Measurement estimates by unit of measure used prior to undergraduate studies

	*Overall* *Mean (SD, range)*	*Inch / foot / yard* *Mean (SD, range)*	*mm / cm / m* *Mean (SD, range)*	t *test* P *value*	*Levene test* P *value*
5-mm mark placement	4.72 (1.83, 2.01–11.69)	5.00 (1.96, 2.01–11.69)	3.93 (1.10, 2.09–6.41)	.011	.190
Absolute error	1.40 (1.19, 0.01–6.69)	1.42 (1.33, 0.01–6.69)	1.34 (0.73, 0.35–2.91)	.746	
1-cm mark placement	10.23 (2.83, 4.82–19.19)	10.68 (2.90, 5.80–19.19)	9.00 (2.25, 4.82–14.76)	.042	.397
Absolute error	2.07 (1.92, 0.09–9.19)	2.15 (2.05, 0.09–9.19)	1.86 (1.56, 0.12–5.18)	.619	
6-mm distance estimation	6.32 (1.60, 4.0–10.0); median 6.0, mode 6.0	6.05 (1.58, 4.0–10.0); median 6.0, mode 5.0	7.06 (1.43, 4.0–10.0); median 7.0, mode 8.0	.028	.597
Absolute error	1.28 (0.99, 0.0–4.0)	1.23 (0.99, 0.0–4.0)	1.44 (1.03, 0.0–4.0)	.473	
9-mm distance estimation	9.43 (1.84, 4.0–12.0); median 10.0, mode 10.0	9.25 (1.89, 4.0–12.0); median 10.0, mode 10.0	9.94 (1.61, 7.0–12.0); median 10.0, mode 10.0	.202	.480
Absolute error	1.47 (1.17, 0.0–5.0)	1.43 (1.25, 0.0–5.0)	1.56 (0.96, 0.0–3.0)	.706	

## DISCUSSION

This study presents the results of the first known length estimation task performed by surgeons. Despite substantial training and surgical experience, many attending surgeons and surgical trainees failed to produce accurate estimates of short distances. Specifically, half of the participants demonstrated an error of more than 20% when placing marks at a 5-mm distance, and half had an error of more than 15% when placing marks at a 1-cm distance. Notably, the range of estimates for the 5-mm distance estimation task was 2.01 to 11.69 mm, and for the 1-cm task, the range was 4.82 to 19.19 mm. This substantial variability not only has potential clinical ramifications but also suggests that a trainee and an attending could have wide disagreement on tasks communicated as estimated distances. Training level was not found to improve estimates, and the use of the metric system of units prior to undergraduate studies was associated with poorer performance on the estimation tasks. These results suggest that the ability to estimate distances is not innate to surgeons, and despite pursuing a significant amount of training, the skills necessary to perform this task are underdeveloped.

The clinical implication of poor distance estimating ability is admittedly unclear, and the present study was not designed to correlate distance estimating ability to clinical outcomes. However, given that suture spacing has been correlated to clinical outcome, one may hypothesize that the ability to accurately estimate the placement of sutures may influence clinical results as well. The STITCH trial demonstrated a reduction in incisional hernia rate from 21% to 13% when small bites (5 mm by 5 mm) were used for fascial closure compared to large bites (1 cm by 1 cm) [3]. Although the results of the STITCH trial provide a widely applicable and simple intervention, the ability to translate the technique to clinical practice depends heavily on the ability to estimate suture placement with accuracy. As demonstrated in our study, there exist surgeons who overestimate a distance of 1 cm to over 19 mm, and these surgeons may place their intended 5-mm sutures at a true spacing closer to 1 cm. Similarly, the participant who estimated a 1-cm distance at 4.82 mm may place their intended 5-mm suture very close to the edge of fascia. Notably, the method of ensuring suture spacing is not specified in the STITCH trial; however, the suture length to wound length ratio does suggest that the small-bites group indeed had closer suture spacing.

The importance of intraoperative distance estimation is also seen when cutting tied sutures with an appropriate tail length. The tail length of a cut suture has been shown to impact the integrity of a knot [4], and it is unlikely that a measurement device will be used intraoperatively to ensure appropriate suture tail length. This task is one of the first assigned to new surgical trainees or medical students and may be the first instance of estimate disagreement that trainees experience. Of course, the actual measured length of a suture tail is unlikely to be of significance so long as the tail length is within a satisfactory range.

The purpose of most length estimation tasks in the operating room is not to produce an accurate estimate; instead, the surgeon aims to perform an intervention that will maximize the benefit to the patient. With fascial suture placement, for instance, the tissue quality may dictate the choice of suture placement, and tissue elasticity may alter the visual appearance of the suture's distance from an edge. Nevertheless, deliberate estimation of length in the operating room occurs frequently as instructions are communicated to trainees, and it is important that the attending surgeon and the trainee have a shared expectation for the described length. To facilitate this improved communication, we believe that the development of length estimation skills could be incorporated into surgical training. Of course, the importance of such an educational intervention would be dependent on the clinical ramifications of distance estimation. Nevertheless, it is the opinion of the authors that incongruent estimates occur frequently between trainee and attending surgeons, and improved estimation ability could enhance communication in the operating room. Routt et al report the results of dedicated teaching directed at suturing skills in medical students, showing increased proficiency with independent practice and interval evaluation of suturing ability [5]. We believe that a similar curriculum of dedicated length estimation tasks can be incorporated into the trainee's early education.

This study is limited by the fact that it is performed at a single medical center and has a relatively small number of participants. Despite this fact, trainees and attendings at our institution have received prior training at a variety of facilities throughout the world, and their shared experience in length estimation may be representative of surgeons overall. This study was performed a nonclinical setting, and it is possible that this survey does not represent distance estimation abilities in the operating room. Additionally, the questions on this survey were single-trial estimates of distance, and there is no measurement of precision or repeatability of estimates. We do not assess whether a single individual is consistently producing high or low estimates. Furthermore, we do not assess individual participant's visual acuity, fine motor ability, or other factors that may contribute to the ability to produce distance estimates, and we did not record sex, race, ethnicity, or socioeconomic status to prevent overpartitioning of our limited data set. Despite these limitations, this study represents the first assessment of length estimation by surgeons and draws attention to a limitation in a basic skill that may have clinical consequence.

In conclusion, the estimation of short distances is a task performed frequently in the operating room; however, the accuracy of such estimates has never been formally assessed. In this study, we present the results of a length estimation survey distributed to attending and trainee surgeons at a single institution. We demonstrate that the range of estimates for a 1-cm distance is 4.82 to 19.19 mm, and level of training does not provide any statistically significant improvement in estimation ability. Although this study does not address the clinical implications of poor distance estimates, there is potential for impact to communication in training. Further work to correlate individual surgeon's distance estimation ability to incisional hernia rate would be required to truly delineate the impact of distance estimation on this important surgical complication.

## Author Contributions

**R. Gregory Conway:** Conceptualization, Methodology, Software, Validation, Formal analysis, Investigation, Data Curation, Writing - Original Draft, Writing - Review & Editing, Visualization. **Natalie O'Neill:** Conceptualization, Methodology, Validation, Investigation, Data Curation, Writing - Review & Editing, Visualization. **Jessica Brown:** Methodology, Validation, Formal analysis, Data Curation, Writing - Review & Editing. **Stephen Kavic:** Conceptualization, Methodology, Validation, Investigation, Resources, Writing - Review & Editing, Supervision, Project administration.

## Conflict of interest

The authors have no conflicts of interest to disclose.

## Funding sources

This study was unfunded.

## Appendix A Supplementary data

Appendix A Survey questions. Supplementary material

## Footnotes

Appendix A Supplementary data to this article can be found online at https://doi.org/10.1016/j.sopen.2020.04.001.
