# Supplementary material for: An educated guess — Distance estimation by surgeons
Source: Surg Open Sci. 2020 Apr 30;2(3):113–6. doi: 10.1016/j.sopen.2020.04.001 (PMC8083008; doi:10.1016/j.sopen.2020.04.001)
Supplement: Appendix A — Survey questions. [file mmc1.pdf]

Appendix A

1. What is your current position in the Department of Surgery?
   a. Pre-research Resident (PGY 1 – PGY 2)
   b. Research Resident
   c. Post-research Resident (PGY 3 – PGY 5)
   d. Fellow
   e. Attending

2. How many years of operative experience do you have (including training)?
   a. 1-5
   b. 6-10
   c. 11-15
   d. 16-20
   e. Greater than 20

3. How many laparotomy closures do you participate in per month?
   a. Less than 5
   b. 6 – 10
   c. 11 – 15
   d. 16 – 20
   e. Greater than 20

4. What suture spacing do you use on fascial stitches during laparotomy closure?
   a. Less than 5mm by 5mm
   b. 5mm by 5mm
   c. More than 5mm by 5mm but less than 1cm by 1cm
   d. 1cm by 1cm
   e. Greater than 1cm by 1cm

5. Place a mark on the line below 5mm from the ‘x’.

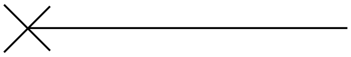

6. On the line below, what is your estimate for the distance between the marks?

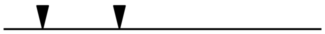

   a. 4mm
   b. 5mm
   c. 6mm
   d. 7mm
   e. 8mm
   f. 9mm
   g. 10mm
   h. 11mm
   i. 12mm

7. On the line below, place a mark 1cm from the ‘x’.

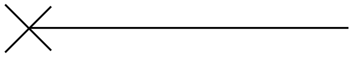

8. On the line below, what is your estimate for the distance between the marks?

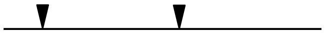

   a. 4mm
   b. 5mm
   c. 6mm
   d. 7mm
   e. 8mm
   f. 9mm
   g. 10mm
   h. 11mm
   i. 12mm

9. Prior to undergraduate studies, which units did you use most frequently to measure length?
   a. Inch / Foot / Yard / Mile
   b. mm / cm / m / km

10. Currently, which units do you use most frequently to measure length?
   a. Inch / Foot / Yard / Mile
   b. mm / cm / m / km
